# Scurvy in a Pediatric Patient Unable to Bear Weight: A Case Report

**DOI:** 10.7759/cureus.38687

**Published:** 2023-05-07

**Authors:** Aleena Jafri, Olayinka Edwards, Puneet Gupta, Rabheh Abdul-Aziz

**Affiliations:** 1 Pediatrics, State University of New York (SUNY) at Buffalo Jacobs School of Medicine and Biomedical Sciences, Buffalo, USA; 2 Family Medicine, Jericho Road Community Health Center, Buffalo, USA; 3 Radiology, John R. Oishei Children's Hospital of Buffalo, Buffalo, USA; 4 Pediatric Rheumatology, State University of New York (SUNY) at Buffalo Jacobs School of Medicine and Biomedical Sciences, Buffalo, USA

**Keywords:** myositis, vascular lesions, neurodevelopmental, speech delay, restricted diet, refusal to walk, non-weightbearing, gum bleeding, vitamin c deficiency, scurvy

## Abstract

Pediatric scurvy is uncommon in the twenty-first century but cases have been reported in children with neurodevelopmental issues and restricted diets. We are reporting a two-year and nine-month-old boy who had a coronavirus disease (COVID) infection and then presented with a refusal to walk. By careful history-taking, he was found to have a restricted diet, speech delay, and gum bleeding suggestive of scurvy, which was confirmed by extremely low levels of ascorbic acid. In this case, the diagnosis of scurvy was established before establishing the diagnosis of neurodevelopmental delay. Treatment with ascorbic acid resulted in a remarkable improvement in his symptoms. This case highlights the importance of collecting a thorough history, connecting exam findings to the history, and including scurvy in differential diagnoses for the presentation of inability to bear weight.

## Introduction

Up to 90% of vitamin C, an essential vitamin for humans, is consumed in the form of fresh fruits and vegetables; thus, insufficient ingestion or poor enteric absorption can lead to deficiency [1]. Furthermore, it is heat-sensitive so common methods of food preparation diminish the amount of utilizable vitamin C [1]. It is excreted renally, so serum levels are heavily dependent on recent intake [1]. Vitamin C is an enzymatic cofactor in collagen synthesis, a ubiquitous protein that lends tensile strength to the walls of vasculature and the basement membrane zone separating the epidermis from the dermis. Cases of deficiency lead to derangement of this process and the production of abnormal collagen, causing the main clinical manifestations [2].

Scurvy, or severe chronic vitamin C deficiency, is an easily preventable clinical syndrome rarely seen in the developed world but most commonly associated with socioeconomic status and food insecurity [2]. In the pediatric world, most cases are accounted for by the presence of neurobehavioral disorders or extremely restricted diets [3]. Symptoms begin to occur when total body stores drop below 300 mg. When clinically manifest, the classic constellation of symptoms is often immediately recognizable, including corkscrew hairs, perifollicular hemorrhage, easy bruising, intraarticular hemorrhage, bone changes, abnormal dentine production and loss of teeth, and gingival bleeding [4]. When seen in conjunction, any of these are highly indicative of vitamin C deficiency [4]. Left untreated, scurvy can be fatal, with case studies reporting causes of death including infection, intracerebral hemorrhage, and hemopericardium [5].

However, because of the wide clinical spectrum of symptoms from musculoskeletal and dental to mucocutaneous to systemic, different presentations of scurvy can mimic a variety of conditions, including autoimmune diseases (vasculitis, juvenile idiopathic arthritis (JIA)), infection (septic arthritis, osteomyelitis, abscess), and malignancy (leukemia, lymphoma, osteosarcoma) [6]. Additional red herrings, such as elevated inflammatory markers, normal nutritional status, and anemia, can lead to unnecessarily complicated, exhaustive workups and delayed diagnosis of scurvy [6]. It should therefore be considered in patients presenting with musculoskeletal complaints, in those with risk factors, as well as in otherwise healthy children, and evaluated with a focused dietary history and comprehensive physical examination [6]. Scurvy has seen a reemergence in high-risk children with autism, mental illnesses, physical disabilities, abnormal dietary habits, gastrointestinal malabsorption, and chronic renal diseases [6,7].

## Case presentation

We report a two-year and nine-month-old boy with a past medical history of speech delay without other developmental delays, who presented to the hospital emergency department (ED) due to one month of lower extremity pain with difficulty ambulating. His parents are Congolese and moved to the United States six years prior. The patient had been evaluated in the ED 18 days prior and discharged home with pain control for suspected transient synovitis. He had been diagnosed with coronavirus disease 2019 (COVID-19) earlier that month. Plain film radiographs of the femurs and pelvis during his initial ED visit found a widened right capital femoral epiphyseal plate, for which orthopedics was consulted but it was not felt to be related to his presenting symptoms. Initial blood work showed normal white blood cells, anemia with a hemoglobin of 9.8 g/dl, and an elevated platelet count of 560,000/uL.

Since then, per the patient’s mother, his symptoms progressively worsened until he was not walking at all nor bearing weight on his lower extremities. His mother denied any noticeable joint swelling, only refusal to bear weight. The patient did not articulate his pain to his parents, as he is largely nonverbal. Per his mother, he had always been a picky eater, and even more so following his COVID-19 infection. Since the age of 14 months, the patient’s very restricted diet comprised almost entirely of flavored yogurt. Mom said sometimes he would see his one-year-old sister eating and try to take a bite but then would not eat. He had not had any abdominal pain, nausea, vomiting, diarrhea, constipation, or blood in the stool. The patient’s mother reported that he would not let his parents brush his teeth, and at a dentist appointment, was noticed to have vascular lesions of the lateral upper gums, which bled with teeth brushing. There was no previous evaluation for speech delay.

**Figure 1:**
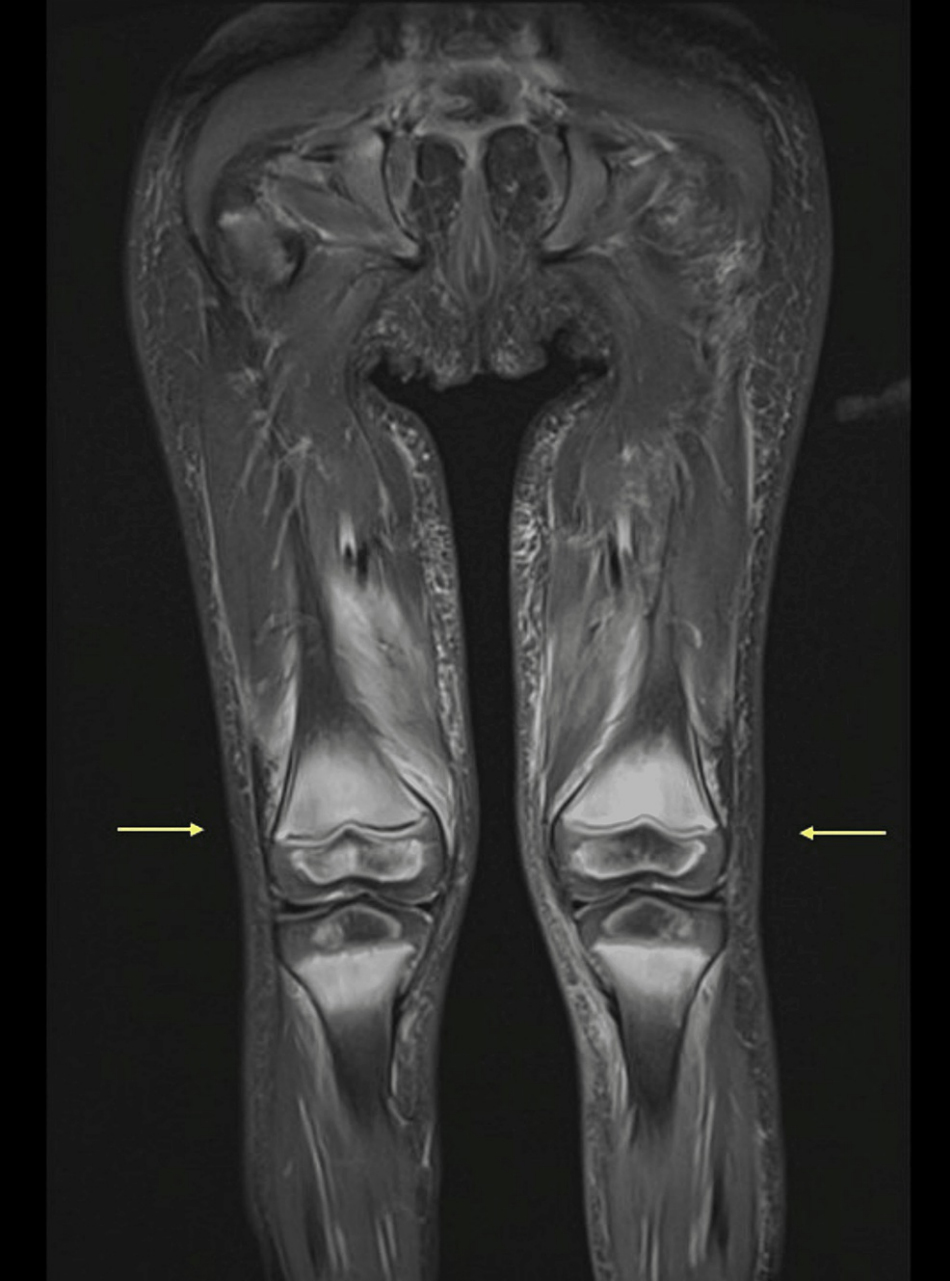
Coronal STIR Coronal STIR demonstrating periosteal enhancement around the femurs (arrows) with no evidence of fluid collection or abscess. STIR: short inversion time inversion recovery

**Figure 2:**
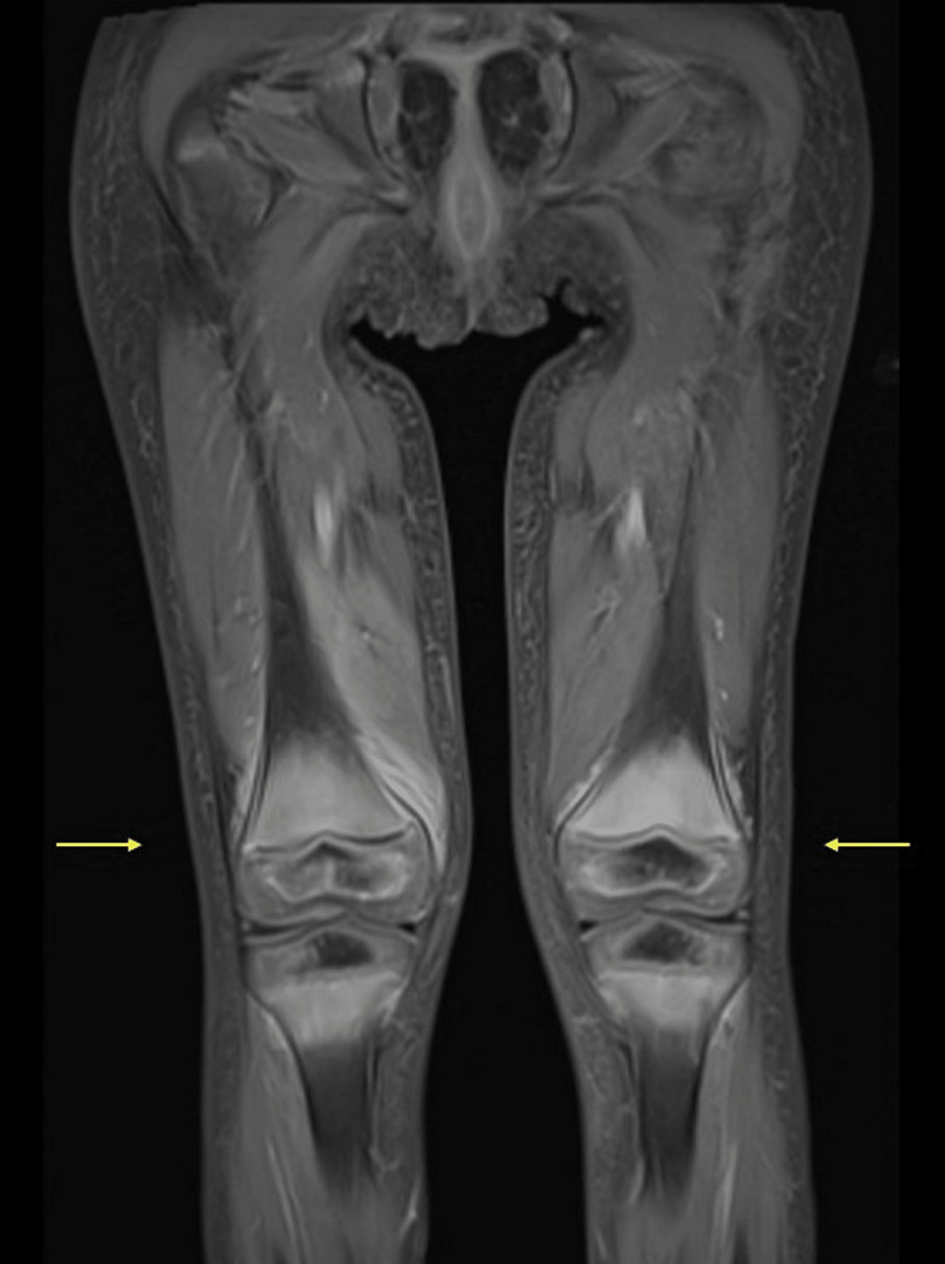
Coronal fat-saturated T1W post contrast Coronal fat-saturated T1W post-contrast demonstrating periosteal enhancement around the femurs (arrows) with no evidence of fluid collection or abscess.

The patient was admitted for further evaluation and treatment. His mother was not aware of any fevers, but the patient was febrile on admission; this resolved within 24 hours. Hip, lumbar spinal, and lower extremity MRIs were obtained. The MRIs revealed extensive enhancing marrow signal abnormality with associated myositis in the pelvis, around the knee joints, and minimally around the ankle joints. There was periosteal enhancement mainly around the femur but no collection or abscess (Figure 1 and Figure 2). There was some physiologic joint fluid but no significant effusion and no synovial enhancement. Mild ascites was noted. Rheumatology was consulted to evaluate for any potential autoimmune disease. His exam showed his weight was 13 kg (25 percentile for his age), with stable vital signs and no rashes. He was laying down in bed with his right leg straight and the left bent at the knee and the hip. He would start crying at any touch of his lower extremities. He refused to stand, and when his mom held him and tried to make him stand, he would not put his feet on the floor. He allowed the full range of motion of the upper extremities without pain on motion and without joint swelling.

In light of the MRI findings and history, particularly the patient’s restricted diet due to picky eating since 14 months of age and his gum bleeding, a suspected diagnosis of scurvy was given. However, broad differential diagnoses, including myositis in association with COVID-19 infection, Multisystem inflammatory syndrome in children (MIS-C) and reactive arthritis in the setting of COVID-19 infection were included as well. Given the absence of any clear inflammatory arthritis by clinical exam and no intraarticular effusion or synovial enhancement in MRI, inflammatory arthritis was excluded. Muscle enzymes showed normal aldolase, aspartate aminotransferase (AST), and alanine transaminase (ALT). He had elevated lactate dehydrogenase (LDH) 352 unit/L and mildly elevated creatine kinase 233 unit/L. Work-up also showed a normal white blood cell count of 6.6 x10^9/L, anemia with hemoglobin 8.1 g/dl, normal platelets 436436 x10^9/L, elevated erythrocyte sedimentation rate (ESR) 50 mm/hr, normal ferritin 22 ng/mL, normal prothrombin time (PT), normal partial thromboplastin time (PTT), elevated D-dimer 3.4 mcg/mL, normal blood urea nitrogen (BUN) and creatinine, low albumin 3.2 g/dL, and normal C-reactive protein (CRP) 8.8 mg/L. Repeat COVID testing was negative, he had COVID antibodies, and he did not fit the criteria for multisystem inflammatory syndrome in children (MIS-C). Upon review of his MRI with radiology and connecting the history of poor diet, gum bleeding, absence of inflammatory arthritis, and absence of juvenile dermatomyositis rashes, we agreed that the finding of myositis in the pelvis, around the knee joints, and around the ankle joints was most likely a manifestation of bleeding within muscles, which is known to occur in patients with scurvy. The vitamin C level was sent.

Symptomatic treatment with nonsteroidal anti-inflammatory medication was initiated while waiting for his vitamin C level result. The patient continued to be non-ambulatory and non-weight-bearing in the hospital. His oral lesions were evaluated by oral surgery, and given their vascular nature, they were felt most likely to be oral manifestations of scurvy, and the biopsy was deferred to outpatient follow-up with oral surgery.

The patient was also arranged to follow up with the feeding clinic, for family assistance with behavior and to work to establish a more well-rounded diet, as well as with the special needs clinic given his speech delay.

After discharge, the patient’s vitamin C level resulted low at <0.1 mg/dL, and he was started on vitamin C at 500 mg daily. After one month of treatment, he started walking and his gum bleeding was resolved. The plan was to repeat the hemoglobin and inflammatory markers but unfortunately, his family lost their insurance and these were not able to be obtained.

## Discussion

A literature review analyzing the clinical features, differential diagnosis, and diagnostic delay of pediatric scurvy found that 127/166 patients with scurvy (76%) had a comorbidity associated with their elevated risk of vitamin C deficiency [6]. These were most commonly reported to be neurological conditions that accounted for 29%, including autism, anorexia, cerebral palsy, and developmental delay [6]. Following this, at 14%, were hematological disorders, including transfusion-related iron overload, recipients of bone marrow transplants, and chronic graft-versus-host disease [6]. The earliest manifestations were nonspecific and constitutional such as fever, malaise, asthenia, irritability, and poor PO intake [6]. In 13 studies and 86 children with scurvy assessed for clinical manifestations, musculoskeletal complaints were described in 92%, most commonly severe lower extremity pain (88%), refusal to walk (73%), and antalgic gait (31%). Mucosal involvement was described in 57% of patients primarily in the form of gingival bleeding (43%) and hypertrophy (27%) with only 5% of children presenting with epistaxis [6].

Our patient presented with an inability to walk, and eliciting a careful history and clinical exam highlighted his speech delay that had not been addressed before and a poor restricted diet limited to flavored yogurt that was also not addressed before. His delay was most likely due to autism spectrum disorder, and he was referred to the developmental clinic for further evaluation. Pediatric scurvy is primarily diagnosed in patients known to have developmental delay, but in our case, scurvy was diagnosed before the patient’s developmental delay was suspected and worked up [8]. A treatment regimen has not been standardized, and pediatric dosing ranges from 100 mg to 1000 mg of vitamin C daily for one month or until remission [9]. It is recommended that doses be divided and distributed throughout the day to avoid saturation of intestinal absorption and renal excretion [9]. Parenteral administration is indicated in patients with vitamin C deficiency due to malabsorption.

One limitation in our case is not being able to repeat the blood work and imaging as the patient's family lost their insurance. At the time of writing this case, the social worker was still working with the family to gain insurance again.

This case emphasizes the need to increase awareness about the presentation of scurvy in pediatric practice. The gravity of this is underscored by evidence revealing that diagnostic hypotheses for various oncological, musculoskeletal infectious, and rheumatic diseases were made even when patients presented with identifiable malnutrition [6]. Most patients will present with unexplained progressive muscle weakness and refusal to walk that will initiate evaluation by multiple sub-specialties, particularly orthopedics and rheumatology. Delay of diagnosis can lead to poor patient outcomes, whether due to extensive and potentially invasive diagnostic workups, hospitalization, and protracted admissions, or the consequences of untreated scurvy itself, many of which may be life-threatening in children. Vitamin C deficiency must be included in the differential for children presenting with musculoskeletal complaints, especially in the setting of known or suspected neurobehavioral disorders, or risk factors for restricted diets. A thorough dietary anamnesis should be elicited, preferably on initial evaluation, and confirmed cases of scurvy should prompt checking for concomitant micronutrient deficiencies.

## Conclusions

This case emphasizes the need to consider scurvy in the differential diagnosis for a child with an unexplained limp, lower extremity weakness, or refusal to walk. Our case highlights the importance of obtaining a dietary history in patients and a careful exam to connect the dots together.
